# Effect of Over-Etching and Prolonged Application Time of a Universal Adhesive on Dentin Bond Strength

**DOI:** 10.3390/polym12122902

**Published:** 2020-12-03

**Authors:** Phoebe Burrer, Hoang Dang, Matej Par, Thomas Attin, Tobias T. Tauböck

**Affiliations:** 1 Clinic of Conservative and Preventive Dentistry, Center for Dental Medicine, University of Zurich, Plattenstrasse 11, 8032 Zurich, Switzerland; h-dang@outlook.com (H.D.); thomas.attin@zzm.uzh.ch (T.A.); tobias.tauboeck@zzm.uzh.ch (T.T.T.); 2 Department of Endodontics and Restorative Dentistry, School of Dental Medicine, University of Zagreb, Gunduliceva 5, 10000 Zagreb, Croatia; mpar@sfzg.hr

**Keywords:** dentistry, dentin, phosphoric acid etching, bonding, universal adhesive, microtensile bond strength

## Abstract

This study investigated the effect of over-etching and prolonged application time of a universal adhesive on dentin bond strength. Ninety extracted human molars were ground to dentin and randomly allocated into nine groups (G1–9; *n* = 10 per group), according to the following acid etching and adhesive application times. In the control group (G1), phosphoric acid etching was performed for 15 s followed by application of the universal adhesive Scotchbond Universal (3M) for 20 s, as per manufacturer’s instructions. In groups G2–5, both the etching and adhesive application times were either halved, doubled, quadrupled, or increased eightfold. In groups G6–9, etching times remained the same as in G2–5 (7.5 s, 30 s, 60 s, and 120 s, respectively), but the adhesive application time was set at 20 s as in the control group (G1). Specimens were then restored with a nanofilled composite material and subjected to microtensile bond strength testing. Bond strength data were statistically analyzed by ANOVA and Tukey’s post-hoc tests (α = 0.05). The relationship of bond strength with etching and adhesive application time was examined using linear regression analysis. Treatment of dentin with halved phosphoric acid etching and adhesive application times (G2) resulted in a significant bond strength decrease compared to the control group (G1) and all other test groups, including the group with halved acid etching, but 20 s of adhesive application time (G6). No significant differences in bond strength were found for groups with multiplied etching times and an adhesive application time of 20 s or more, when compared to the control group (G1). In conclusion, a universal adhesive application time of at least 20 s is recommended when bonding to over-etched dentin.

## 1. Introduction

The achievement of a strong and solid hybrid layer plays an important role in bonding composite to dentin and is still a challenge during adhesive restorative procedures [1,2,3,4,5,6,7,8]. Establishing reliable adhesion to dentin is elaborate due to its high organic and water content, as well as low surface energy and the presence of a smear layer [7]. The subsequently applied polymer composite has to combine good physical, mechanical and biological properties, while matching the dental hard tissue to be restored [9,10]. For hybridization of dentin prior to the application of a resin composite, two adhesive approaches can be distinguished: (1) the etch-and-rinse approach and (2) the self-etch approach [2,7,11]. The etch-and-rinse approach implies complete removal of the smear layer by a separate phosphoric acid etching step, followed by water rinsing and drying of the surfaces prior to application of the adhesive system [11,12,13]. The self-etch approach, however, relies on self-etching acidic monomers instead of phosphoric acid to rather integrate the smear-layer and to simultaneously demineralize and infiltrate dental hard tissue [2,14,15].

In recent years, the use of modern so-called “universal” or “multi-mode” adhesive systems has greatly increased due to their convenient handling. These simplified single bottle adhesives are applicable in the two aforementioned approaches, depending on the clinical situation and the clinician’s preference [16,17,18,19,20,21,22,23]. The composition of universal adhesives includes acidic functional monomers that are able to etch dental hard tissues according to their functional group and establish micro-mechanical and chemical bonding with the tooth surface [5,15,20,24,25] and various restoration materials [6,22,24,26]. Controversial results were found for dentin bond strength when an additional phosphoric acid etching step was carried out prior to the application of universal adhesives, ranging from similar to higher bond strengths compared to the self-etch application mode [20,21,27,28,29,30,31], especially for ultra-mild adhesives [2,4,20].

In order to enhance enamel bonding performance of universal adhesives [7,19,20,21,24], the technically demanding selective enamel etching procedure is often recommended which, however, bears the risk of contaminating neighboring dentin surfaces with phosphoric acid. Furthermore, the etch-and-rinse approach entails the risk of over-etching or over-drying dentin which may result in collapse of collagen fibrils [11,21]. As a consequence, incomplete infiltration of the adhesive [32,33,34,35] and activated endogenous matrix-metalloproteinases degrading exposed collagen in the hybrid layer might lead to reduced bond strength [36,37].

Thus, clinicians face several challenges during the adhesive pretreatment procedure and may frequently be confronted with the issue of accidentally over-etched dentin. Hypothetically, an accidentally over-etched dentin surface might be re-established by deliberately prolonged adhesive application times, possibly leading to complete adhesive infiltration of the excessively demineralized dentin. Therefore, the aim of the present study was to assess the influence of increased phosphoric acid etching and adhesive application times on the bond strength of a universal adhesive in contrast to solely increased acid etching times followed by an adhesive application time as per the manufacturer’s instructions. The null hypothesis proposed that the bond strength to accidentally over-etched dentin cannot be re-established.

## 2. Materials and Methods

### 2.1. Specimen Preparation

Ninety sound human third molars were selected from the department’s collection of extracted teeth (Department of Conservative and Preventive Dentistry, University of Zurich) for this in vitro study. All teeth had been extracted during regular dental therapy, and patients had given their written consent to use the teeth for research purposes. Because the teeth were irreversibly anonymized and could not be tracked back to their donors, this research was exempted from the need to get ethical approval by the local ethics committee (Federal Act on Medical Research involving Human Subjects), and a declaration of non-jurisdiction (BASEC-Nr. 2019-00052) was obtained from the Swiss Ethics Committees on Research.

The extracted teeth were then cleaned [38] and stored in 0.5% chloramine-T solution [8,27,39]. In order to facilitate the subsequent use, the root tips were glued perpendicular to the center of a holder for scanning electron microscopes (Wenka, Karl Wenger SA, Courgenay, Switzerland) with superglue (Superglue No. 1733-2000, Renfert, Hilzingen, Germany) and embedded in self-curing acrylic resin (Paladur, Heraeus Kulzer, Hanau, Germany). Then, the tooth crowns were cut parallel to the occlusal surface with a low-speed diamond saw (IsoMet, Buehler, Lake Bluff, IL, USA) and ground with a polishing machine (Planopol-2, Struers GmbH, Ballerup, Denmark) at low speed (150 rpm) under permanent water cooling with 180-grit silicon carbide paper (Buehler-Met II, Buehler, Lake Bluff, IL, USA) until a complete dentin layer was exposed. This surface was checked under a stereomicroscope (Stemi 1000, Carl Zeiss AG, Oberkochen, Germany) at 25× magnification to ensure that no enamel remnants were left or pulp tissue exposure had occurred in the central part of the tooth.

### 2.2. Acid Etching Treatment and Adhesive Application Procedure

**Figure 1:**
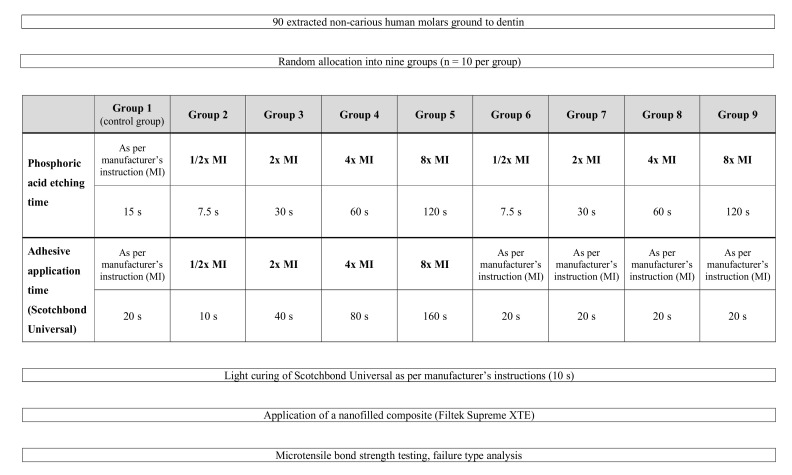
Experimental design.

The experimental design is described in Figure 1, and the composition of the main materials used in the current study is provided in Table 1. The teeth were randomly allocated into nine groups (*n* = 10 per group) and treated as follows:

**Group G1 (control group):** Application of the universal adhesive Scotchbond Universal (3M, St. Paul, MN, USA; lot no.: 636396) was performed strictly following the manufacturer’s instructions for use. For this purpose, a phosphoric acid etchant gel (35% phosphoric acid Ultra Etch, Ultradent Products, South Jordan, UT, USA) was applied on the dentin surface for 15 s [39,40] according to the manufacturer’s instructions, followed by water-rinsing until the etchant gel was completely removed, and careful air-drying of the dentin surface. Scotchbond Universal was then actively applied with a microbrush for 20 s, gently dispersed with air for 5 s to evaporate the solvent and light cured for 10 s with an LED light-curing unit (Bluephase G2, Ivoclar Vivadent, Schaan, Liechtenstein). The output irradiance of the curing unit (1315 mW/cm^2^) was measured by using a calibrated FieldMax II-TO power meter (Coherent, Santa Clara, CA, USA) and verified at regular intervals [41].

**Group G2 (halved acid etching and adhesive application times):** Bonding procedure as described in group 1, however, with halved phosphoric acid etching time (7.5 s) and halved adhesive application time (10 s).

**Group G3 (doubled acid etching and adhesive application times):** Bonding procedure as in group 1, but with an acid etching time of 30 s and adhesive application for 40 s.

**Group G4 (quadrupled acid etching and adhesive application times):** Bonding procedure as in group 1, however, with acid etching for 60 s and an adhesive application time of 80 s.

**Group G5 (eightfold acid etching and adhesive application times):** Bonding procedure as in group 1, however, with phosphoric acid etching for 120 s and adhesive application for 160 s.

**Groups G6–9:** In these groups, the phosphoric acid etching time was either halved (G6), doubled (G7), quadrupled (G8), or increased eightfold (G9) as described in groups 2–5, whereas the application time of Scotchbond Universal was set at 20 s, as specified by the manufacturer.

### 2.3. Microtensile Bond Strength Test

Specimens were subjected to a previously described standard bond strength testing procedure to determine microtensile bond strength [42,43]. In brief, all specimens were sectioned longitudinally in two directions using a diamond cut-off wheel (M1D10, Struers GmbH, Ballerup, Denmark) in a precision cutting machine with constant water irrigation to obtain rectangular sticks (approximately 0.9 × 0.9 mm^2^) from the center of each tooth. Edge lengths of sticks were measured with a digital caliper (Kisling, Zurich, Switzerland), and the mean cross-sectional bonding area amounted to 0.845 mm^2^. All specimens were attached with superglue (Superglue No. 1733–2000, Renfert, Hilzingen, Germany) at both ends to a sandblasted (50 µm aluminum oxide) microtensile bond strength jig (produced according to a custom-made template by Wenka, Karl Wenger SA, Courgenay, Switzerland) and loaded under tension in a universal testing machine (Zwick Roell Z010, ZwickRoell, Ulm, Germany) with a crosshead speed of 1 mm/min until failure. The load at failure (N) divided by the bonding area (mm^2^) resulted in the microtensile bond strength in MPa.

### 2.4. Assessment of Failure Modes

After microtensile bond strength testing, all sticks were examined for failure mode analysis using an optical microscope (Stemi 1000, Carl Zeiss AG, Oberkochen, Germany) at 25× magnification. Failure modes were classified as adhesive (between dentin and composite buildup), cohesive (within dentin or within the composite buildup) or mixed failure (both adhesive and cohesive).

### 2.5. Statistical Analysis

The microtensile bond strength of specimens that failed prior to testing (pre-test failures) was set to 0 MPa [44]. Descriptive statistics such as mean, standard deviation, minimum, median and maximum values were calculated for each group. Assumptions of the parametric approach (homogeneity of variance and normality) were checked using residual plots and no violations were found. Data were statistically analyzed between groups using one-way analysis of variance (ANOVA) followed by pairwise HSD post-hoc tests corrected for multiple comparisons according to Tukey. Linear regression analyses were performed to test the relationship between microtensile bond strength and etching time/adhesive application time. An additional linear regression was performed for the relationship between failure mode and etching time/adhesive application time. Statistical analyses were performed using the open-source statistical software R (R version 3.2.2., R Development Core Team, R Foundation for Statistical Computing, Vienna, Austria) [45]. The level of significance was set to ± = 0.05.

## 3. Results

### 3.1. Microtensile Bond Strength

**Figure 2:**
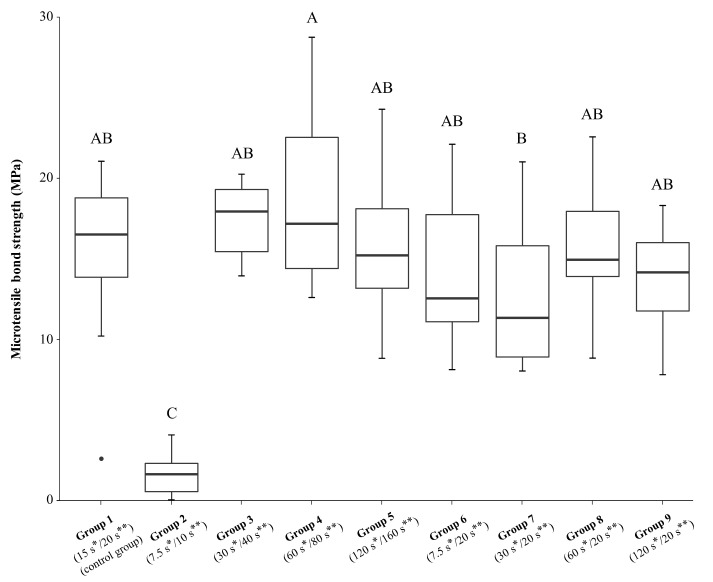
Microtensile bond strength (MPa) of dentin–composite interfaces after different phosphoric etching and adhesive application times. Groups marked with different letters are significantly different from each other at the 0.05 level. The boxplots show the medians (black lines) with 25% and 75% quartiles (boxes); the whiskers represent 1.5× interquartile range (IQR), or minima and maxima of the distribution if below 1.5× IQR. The outlier in group 1 is depicted as dot. * Phosphoric acid etching time; ** adhesive application time.

Descriptive statistics of the microtensile bond strength of all groups after the different phosphoric etching and adhesive application times are given in Table 2. As shown in Figure 2, adhesive pretreatment of the dentin surface with halved etching time and halved adhesive application time resulted in a significant decrease in bond strength (G2: 1.51 ± 1.27 MPa) compared to the control group (G1: 15.14 ± 5.46 MPa) and all other test groups, including the group with halved etching time but 20 s of adhesive application (G6: 14.16 ± 5.01 MPa). No significant differences in dentin bond strengths were observed between the groups with doubled (G3: 17.40 ± 2.33 MPa), quadrupled (G4: 18.84 ± 5.50 MPa) or eightfold increased etching and adhesive application times (G5: 15.88 ± 4.58) and the control group. Within test groups in which only the etching time was changed but the application time of the adhesive remained 20 s (G6–9), no significant bond strength differences could be detected. A significant difference (*p* = 0.044) could be observed between group 4 with quadrupled etching and adhesive application times, and group 7 with doubled etching time and application time of Scotchbond Universal as per manufacturer’s instructions (20 s). Test groups 3–5 with increased etching times and subsequently prolonged adhesive application times showed higher bond strength values, albeit not being statistically significant, compared to test groups 7–9 with increased acid etching times but constant adhesive application time of 20 s. Regression analysis showed no linear relationship between microtensile bond strength and etching time/adhesive application time (*p* > 0.05).

### 3.2. Failure Modes

**Figure 3:**
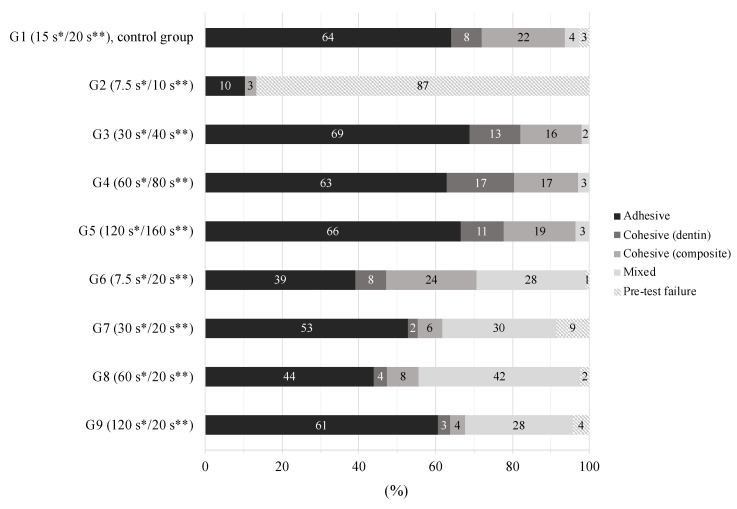
Distribution of failure modes per group, given in percentage (%). * Phosphoric acid etching time; ** adhesive application time.

Failure mode distributions of the experimental groups are given in Figure 3, showing that the predominant failure mode of all groups was adhesive failure. The percentage of pre-test failures was highest in the group with halved acid etching and halved adhesive application time (G2). No linear relationship was found between failure mode and etching time/adhesive application time (*p* > 0.05).

## 4. Discussion

Given that dentin surfaces can easily be over-etched accidentally during adhesive pretreatments, this study hypothesized that an over-etching of dentin might be compensated by a prolonged adhesive infiltration time. Interestingly though, results indicate that an adhesive application time of 20 s during bonding procedures is sufficient to re-establish bond strength, even when excessive over-etching of dentin has occurred. Hence, the null hypothesis was rejected.

Ideally, adhesives should reach the bottom of the demineralized dentin layer and entirely infiltrate dentin collagen fibrils in order to prevent nanoleakage and a decrease of bond strength [1]. Previous studies found a direct correlation between extended acid etching time and demineralization depth, resulting in a poorly infiltrated hybrid layer [33,46], increased surface roughness [35] and reduced bond strength [34,47]. However, our results indicate that even with multiplied acid etching times and an application time of 20 s of Scotchbond Universal as per the manufacturer’s instructions (G7–9), similar dentin bond strengths can be achieved as in groups with both multiplied acid etching and adhesive application times (G3–5). This is also in line with a study of Paul et al. [32], in which no significant differences in bond strength were found regardless of a performed dentin over-etching up to 60 s but constant adhesive infiltration time. Nevertheless, it should be noted that in the present study, groups with extended adhesive application time (G3–5) showed slightly, yet not statistically significantly improved dentin bond strengths compared to groups with an application time of 20 s (G7–9).

An explanation why an adhesive application time of 20 s might be sufficient for re-establishing dentin bond strength after excessive phosphoric acid etching might be found when drawing attention to the interaction of acid etching effects on the dentin structure. It has been reported that remaining hydroxyapatite exhibits a buffering effect on the acid reaction with dentin [46,48] and that acid penetration might be decreased by the outward directed dentin liquid flow due to diluting effects [49]. In the present study, a combination of the factors application mode, viscosity and acid concentration of the applied phosphoric acid etchant is likely to have had an impact on the resulting demineralization depth according to the Nernst diffusion layer hypothesis [50,51]. Transferred to our study, it describes the immediate formation of a semi-static layer between the phosphoric acid solution and the dentin surface, where diffusion occurs via a concentration gradient [50]. The applied phosphoric acid could have been used up faster and the demineralization process reached saturation after shorter acid etching times than the excessively chosen ones of the present study [51]. These characteristics could limit the demineralization process and ensure an entire adhesive infiltration during 20 s, even if prolonged etching periods have occurred.

Another aspect considers the high wettability of Scotchbond Universal promoting the infiltration process due to the contained 2-hydroxylethyl methacrylate (HEMA). Moreover, by applying the adhesive with active rubbing motions, fresh monomers are constantly provided to enhance adhesive infiltration. Positive effects of altered application modes (active, prolonged or multiplied application) on the polymer–dentin bond strengths have been reported for universal adhesives in recent studies [8,24,25,30,39] and can be attributed to the better infiltration of monomers into decalcified dentin [8,38]. The fact that the low bond strengths of the group with halved acid etching time and halved adhesive application time (G2) could almost be raised to the level of the control group (G1) by 20 s of adhesive application time (G6) emphasizes the importance of strict adherence to the minimum recommended application time. The low percentage of pre-test failures in group 6 compared to group 2 further supports these considerations. The overall occurrence of mainly adhesive fractures in the present study is also in accordance with other studies examining dentin bond strengths [25,28,39,42].

Recent studies depicted the dependence of pH-value of the applied universal adhesive on the bonding performance and emphasized the importance of their knowledge to the practitioner [21,39]. Higher bond strengths were observed for mild adhesives compared to adhesives with lower pH-values [8,52]. The classification of Scotchbond Universal as mild or ultra-mild adhesive (pH = 2.7) might therefore also possibly explain the sufficient bond strengths even after excessive acid contamination of dentin.

A limitation of the present study might be the investigation of only one phosphoric etchant. Etching solutions vary in viscosity and pH-level, so that the findings of the present study cannot be generalized. The present report tested a specific universal adhesive and a single composite resin. The use of other (universal) adhesives with distinct formulations might obtain different results. In fact, many variables could have an influence on material behavior, such as the composite density [53], the presence of nanofillers [9] and the dentin surface morphology [38]. These factors could alter the bonding interface, thus having a possible effect on final results. Concerns can also be raised about the long-term stability of the polymer–dentin interface after excessive acid etching. Furthermore, the present study was conducted in vitro, and results cannot be directly assigned to a clinical environment.

## 5. Conclusions

Based on the results of the present in vitro study, it can be concluded that in case of accidental over-etching of dentin, an adhesive application time of at least 20 s is mandatory to achieve sufficient dentin bond strength for the tested universal adhesive. Future (clinical) studies are needed in order to confirm the results of the present study and to further investigate the (long-term) interaction of over-etched dentin and following polymer application protocols.

## Figures and Tables

**Table 1:** Manufacturers’ information about the main materials used in the present study.

Material	Composition	Lot No.	Manufacturer
Scotchbond Universal	10-MDP ^1^, dimethacrylate resins, HEMA ^2^, methacrylate-modified polyalkenoic acid copolymer, filler, ethanol, water, initiators, silane	636396	3M, St. Paul, MN, USA
Filtek Supreme XTE	Matrix: Bis-EMA ^3^, Bis-GMA ^4^, PEGDMA ^5^, TEGDMA ^6^, UDMA ^7^ Filler: non-agglomerated/non-aggregated silica (20 nm) and zirconia (4–11 nm) fillers, aggregated zirconia/silica cluster filler (average cluster particle size: 0.6–10 µm), filler content: 78.5 wt%	N850885	3M, St. Paul, MN, USA

^1^ 10-MDP: 10-methacryloyloxydecyl dihydrogen phosphate; ^2^ HEMA: 2-hydroxylethyl methacrylate; ^3^ Bis-EMA: ethoxylated bisphenol-A-glycidyl methacrylate; ^4^ Bis-GMA: bisphenol-A-glycidyl-dimethacrylate; ^5^ PEGDMA: poly(ethylene glycol) dimethacrylate; ^6^ TEGDMA: triethylene glycol dimethacrylate; ^7^ UDMA: urethane dimethacrylate.

**Table 2:** Descriptive statistics of the microtensile bond strength (MPa) of all tested groups.

Group	Mean	SD	Minimum	Median	Maximum
G1 (15 s */20 s **), control group	15.14	5.46	2.54	16.46	21.01
G2 (7.5 s */10 s **)	1.51	1.27	0.00	1.58	4.02
G3 (30 s */40 s **)	17.40	2.33	13.88	17.88	20.21
G4 (60 s */80 s **)	18.84	5.50	12.55	17.13	28.69
G5 (120 s */160 s **)	15.88	4.58	8.78	15.16	24.24
G6 (7.5 s */20 s **)	14.16	5.01	8.08	12.49	22.05
G7 (30 s */20 s **)	12.68	4.84	7.99	11.28	20.97
G8 (60 s */20 s **)	15.82	4.03	8.79	14.89	22.51
G9 (120 s */20 s **)	13.84	3.23	7.76	14.11	18.25

* Phosphoric acid etching time; ** adhesive application time. SD: standard deviation.
